# Exploring the Effects of Geographical Origin on the Chemical Composition and Quality Grading of *Vitis vinifera* L. cv. Chardonnay Grapes

**DOI:** 10.3390/molecules22020218

**Published:** 2017-01-31

**Authors:** Joanna M. Gambetta, Daniel Cozzolino, Susan E. P. Bastian, David W. Jeffery

**Affiliations:** 1 School of Agriculture, Food and Wine, Waite Research Institute, The University of Adelaide, PMB 1, Glen Osmond, SA 5064, Australia; joanna.gambetta@adelaide.edu.au (J.M.G.); sue.bastian@adelaide.edu.au (S.E.P.B.); 2 School of Medical and Applied Sciences, Central Queensland Innovation and Research Precinct, Central Queensland University, Bruce Highway, North Rockhampton, QLD 4701, Australia; d.cozzolino@cqu.edu.au

**Keywords:** Chardonnay, geographical indication, volatile composition, elements, multiple factor analysis

## Abstract

The relationship between berry chemical composition, region of origin and quality grade was investigated for Chardonnay grapes sourced from vineyards located in seven South Australian Geographical Indications (GI). Measurements of basic chemical parameters, amino acids, elements, and free and bound volatiles were conducted for grapes collected during 2015 and 2016. Multiple factor analysis (MFA) was used to determine the sets of data that best discriminated each GI and quality grade. Important components for the discrimination of grapes based on GI were 2-phenylethanol, benzyl alcohol and C_6_ compounds, as well as Cu, Zn, and Mg, titratable acidity (TA), total soluble solids (TSS), and pH. Discriminant analysis (DA) based on MFA results correctly classified 100% of the samples into GI in 2015 and 2016. Classification according to grade was achieved based on the results for elements such as Cu, Na, Fe, volatiles including C_6_ and aryl alcohols, hydrolytically-released volatiles such as (*Z*)-linalool oxide and vitispirane, pH, TSS, alanine and proline. Correct classification through DA according to grade was 100% for both vintages. Significant correlations were observed between climate, GI, grade, and berry composition. Climate influenced the synthesis of free and bound volatiles as well as amino acids, sugars, and acids, as a result of higher temperatures and precipitation.

## 1. Introduction

Place of origin has an important influence on the style, quality and prestige of regional produce such as wine. Protected “Geographical Indications” (GI) have thus arisen as a way to identify goods that originate from a particular region “where a given quality, reputation or other characteristic of the product is essentially attributable to its geographical origin” [1]. GI are used in recognition of the influence of local factors on the characteristics of products that can help differentiate them in the global market [2]. Wine provenance and quality begins with the grapevines that are grown in a multitude of regions around the world, some of which are better suited to certain varieties than others. Climate and vineyard characters such as geology and soil affect vine phenology, and vine water and mineral status, modifying grape chemical composition [3] and enabling discrimination of fruit from different regions [4,5,6,7]. Knowledge of the specific composition of the grapes originating from different GI would allow the characterisation, authentication and valorisation of each GI.

The presence of major and trace minerals in the berry directly impacts yeast behaviour and modifies wine sensory profile and quality [8]. Concentrations in the berry depend on the availability of elements such as Fe, Zn and Mg in the soil, which are modulated by agricultural practices, particularly irrigation and fertilisation [9]. A number of studies have demonstrated that wine mineral profile is highly correlated to region and soil type, leading to the use of mineral composition to discriminate between wines from different origins [4]. However, few studies have been performed on grapes. Cugnetto et al. [10] were able to discriminate between grapes from the Alpine and Langhe regions based on their Ba, Mn, Si, Sr, and Ti concentrations, and Protano and Rossi [11] classified grapes according to the composition of the soil of origin using Ba, Rb, and Sr as markers. However, both of these studies dealt with viticultural areas with distinctly different soil profiles. In fact, Cugnetto et al. [10] were unable to discriminate between regions that were closer together based solely on berry element composition.

Conceptual representations exist for wines originating from different geographical origins [12]; Cabernet Sauvignon wines from California and Australia, Riesling wines from Germany, and Sauvignon Blanc wines from New Zealand, amongst others, have been shown to possess distinct and typical sensory characteristics that allow them to be recognised and discriminated from wines produced in other regions [13,14,15,16,17]. These typical sensory representations correspond to the biochemical profiles, and particularly the volatile aroma compositions of each set of wines, where clear correlations exist between the presence of certain aroma notes, for example floral, citrus and lime, and compounds such as monoterpenoids [18]. The responsible secondary metabolites are produced in the berry through a series of biosynthetic pathways which are modulated by viticultural practices, and particularly, by climatic phenomena [19,20]. This knowledge has led to investigations of the link between volatile composition and GI in order to classify samples according to their origin [13,21,22,23,24]. Aroma compounds are found in free and bound forms in the berry. Free forms include alcohols, aldehydes, acetates and isoprenoids [25], and bound forms (in terms of being hydrolytically-releasable) involve aglycones such as C_13_-norisoprenoids, aliphatic and aromatic alcohols, shikimic acid metabolites, and monoterpenoids linked to sugars [26].

The successful classification of grapes and wines according to their origin is based on data sets that are intrinsically multivariate, and often combines results from various analytical techniques [4]. Chemometric tools are needed to determine the most important factors that need to be measured to predict origin, as well as to recognise patterns in the data and develop classification models [27], and techniques such as principal component analysis (PCA), multiple factor analysis (MFA), cluster analysis (CA), discriminant analysis (DA), and partial least squares (PLS) regression have commonly been used. PCA is an exploratory technique used to reduce the dimensionality of a data set [27]. MFA is similarly exploratory and allows the simultaneous analysis of multiple data sets, structured in groups, in which each group of variables is weighted [28]. DA is capable of classifying samples into pre-established categories and PLS regression is mainly used to relate blocks of variables measured on sets of objects [29,30,31].

In this study we used chemometric approaches to explore the effects of geographical origin on the chemical composition of Chardonnay grapes obtained from seven GI in South Australia during two vintages, and thereby determined the variables capable of discriminating between the regions of origin. Grape allocation grade was then used to try to pinpoint the chemical variables driving the grades assigned by winemakers in order to investigate objective measures of Chardonnay grape quality.

## 2. Results and Discussion

### 2.1. Understanding Regional Effects on Grape Composition

Grape samples were collected at commercial maturity during the 2015 and 2016 vintages from different GI across South Australia spanning the Adelaide Hills (ADL), Barossa Valley (BV), Clare Valley (CV), Eden Valley (EV), McLaren Vale (MV), Langhorne Creek (LC) and the Riverland (RVL). Measurements of basic chemical parameters (TSS, TA and pH), 12 elements, 28 free and 29 conjugated volatile compounds and 19 amino acids were performed on the various grape samples (Supplementary Tables S1–S5). Examination of these compositional variables by two-way ANOVA (using region and vintage as the explaining variables) showed statistically significant interactions (*p* < 0.05) between both variables for most of the evaluated parameters. Significant differences were found for most compounds measured between both years with the exception of: Na, S, Fe, B, Zn, P, serine, histidine, threonine, leucine, phenylalanine, arginine, lysine, isoleucine, the free volatiles isoamyl acetate, hexanoic acid, (*E*)-3-hexen-1-ol, 2-ethyl-1-hexanol, linalool, (*Z*)-linalool oxide, hexanal and the conjugated volatiles 2,6-dimethyl-7-octene-2,6-diol, 2,6-dimethoxyphenol and decanoic acid. Additionally, PCA (results not shown) of the combined 2015 and 2016 analytical data revealed a clear clustering of samples according to vintage, so it was decided to treat both vintages separately. Vintage effects are common given the large but variable influences that seasonal changes have on berry metabolite composition [32,33], as climate has been shown to have a greater effect on fruit composition than soil and cultivar [34].

#### 2.1.1. Classification According to Origin Using Multiple Factor Analysis of Analytical Variables

**Figure 1:**
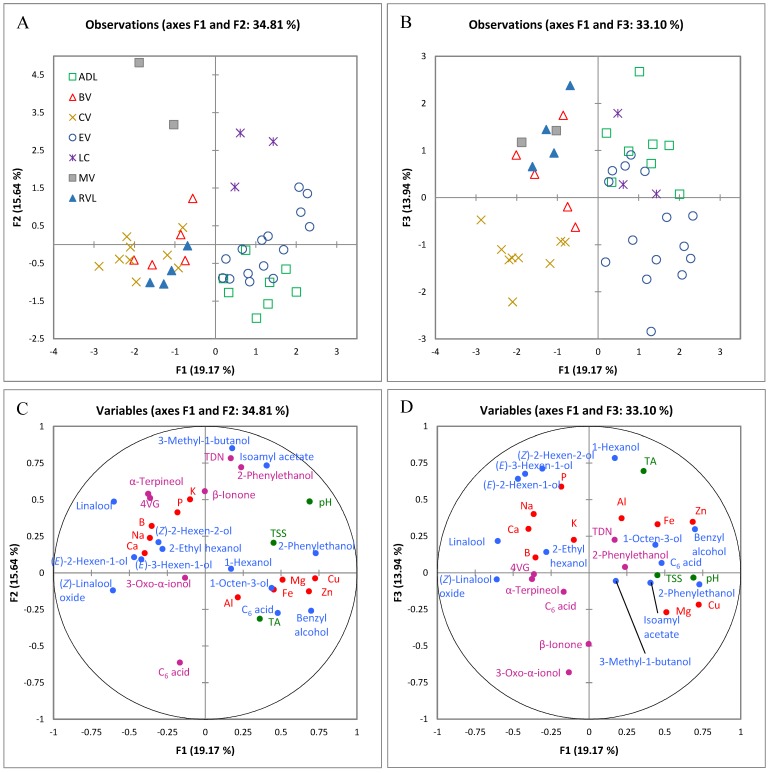
Multiple factor analysis (MFA) of elements, free and bound volatiles and basic chemistry data for all seven Geographical Indications (GI) in 2015, showing the scores projected on F1 and F2 (**A**) and F1 and F3 (**B**) for each GI, and loadings of variables used in the analysis on F1 and F2 (**C**) and F1 and F3 (**D**) (basic chemistry in green, elements in red, free volatiles in blue and bound volatiles in violet). ADL, Adelaide Hills; BV, Barossa Valley; CV, Clare Valley; EV, Eden Valley; LC, Langhorne Creek; MV, McLaren Vale; RVL, Riverland. Abbreviations (e.g., TSS, 4VG, TDN) are the same as specified in the text.

**Figure 2:**
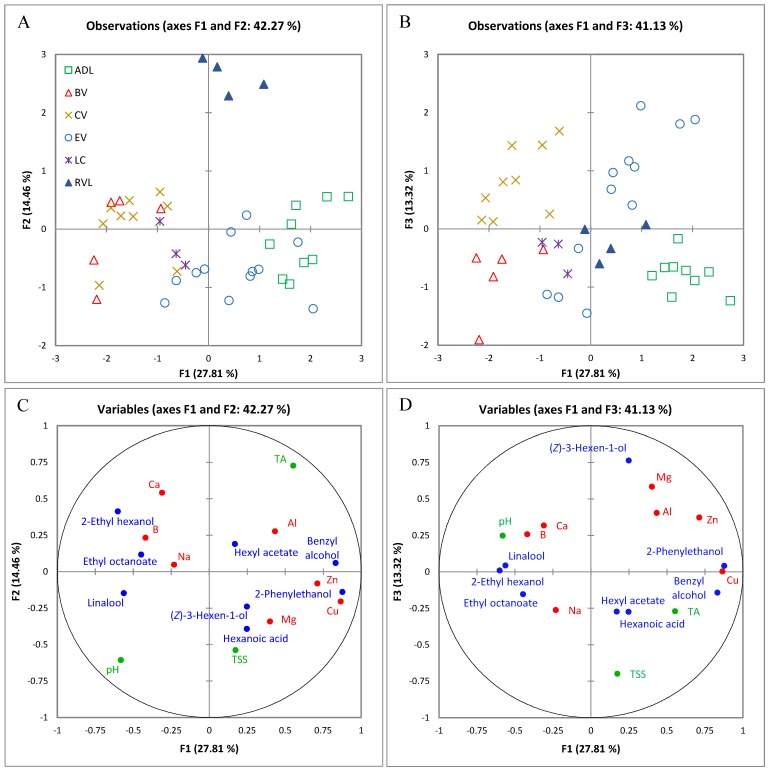
MFA of elements, free volatiles and basic chemistry data for all six GI in 2016, showing the scores projected on F1 and F2 (**A**) and F1 and F3 (**B**) for each GI, and loadings of variables used in the analysis on F1 and F2 (**C**) and F1 and F3 (**D**) (basic chemistry in green, elements in red and free volatiles in blue). ADL, Adelaide Hills; BV, Barossa Valley; CV, Clare Valley; EV, Eden Valley; LC, Langhorne Creek; RVL, Riverland. Abbreviations (e.g., TSS, TA) are the same as specified in the text.

To elucidate the relationship between grape chemical composition and the region of origin, MFA was applied to significantly different compositional variables (using GI as the explaining variable). MFA is a *k*-table methodology that allows the simultaneous analysis of multiple data sets acquired on the same group of samples, and unlike other methodologies, it ensures that no single set dominates the common solution. Only data sets with RV coefficients superior to 0.6 were retained, and MFA was recalculated for 2015 using the data sets corresponding to elements, free volatiles, basic chemistry and bound volatiles (Figure 1) and for 2016 using elements, free volatiles, and basic chemistry (Figure 2).

#### 2.1.2. Differentiating Variables

The first three dimensions of the MFA plot corresponding to the 2015 vintage accounted for 49% of the total variance (Figure 1). Four groups of samples were observable in the F1/F2 plot (Figure 1A). Group 1, comprising ADL and EV samples, was located in the lower right quadrant of F1/F2, group 2 containing BV, CV, and RVL samples was located in the lower left quadrant of F1/F2, and two groups consisting separately of LC and MV samples, were located on the positive side of F2. Inclusion of F3 (Figure 1B) allowed the separation of CV samples from the rest of group 2 along the F3-axis (lower left quadrant of F1/F3, Figure 1B). Examination of F1 loadings (Figure 1C) revealed that the separation between group 1 and 2 was due to a higher pH and higher concentrations of Cu, Zn, free 2-phenylethanol and benzyl alcohol, and lower concentrations of free (*Z*)-linalool oxide and linalool in the ADL and EV samples (Supplementary Tables S1, S2, and S3, Supplementary Figure S1) to the right along F1. Notably, all samples on the left side of F1 (Figure 1A) originated from regions with high night time temperatures during the berry ripening period in January and February (Supplementary Table S6), which correlated with lower concentrations of 2-phenylethanol and benzyl alcohol (r = −0.49 and r = −0.64 respectively, *p* < 0.001) and higher contents of linalool (only January, r = 0.69, *p* < 0.0001). Benzyl derivatives are derived from l-phenylalanine and are formed through a coupled decarboxylation and oxidation reaction (2-phenylethanol) or through deamination of phenylalanine into (*E*)-cinnamic acid and subsequent oxidation (benzyl alcohol) [25]. These compounds have been reported to be present at higher concentrations in Glera grapes at véraison, decreasing thereafter as maturity progresses [35]. As observed by Alessandrini et al. [35], benzyl derivatives were also the most abundant in our study for the two sites with the highest altitudes, ADL and EV.

The presence of certain trace elements has been used in several studies to determine the geographical origin of wines and grapes, as they reflect the geochemistry of the soil the vines were grown in [4]. Cu and Zn were most abundant in ADL and EV (Figure 1A,C, Supplementary Table S2, Supplementary Figure S1), which contributed to their separation from the rest of the sites. Separation of LC and MV from group 1 and 2 along F2 (Figure 1A) was driven by higher contents of the free volatiles isoamyl acetate and 3-methyl-1-butanol, and bound 1,1,6-trimethyl-1,2-dihydronaphthalene (TDN) and 2-phenylethanol (Figure 1C, Supplementary Tables S3 and S5) and lower concentrations of bound hexanoic acid. MV had both the highest growing degree day (GDD) values and night time temperatures in 2015 (Supplementary Table S6), contributing to the formation of free 3-methyl-1-butanol and isoamyl acetate, and bound TDN and 2-phenylethanol, along with the degradation of hexanoic acid (bound). Similarly, higher formation of 3-methyl-1-butanol has been reported in Glera berries in warm sites compared to cool sites in the Conegliano-Valdobbiadene appellation [35]. A lower concentration of the C_6_ compounds 1-hexanol, (*E*)-3-hexen-1-ol, (*Z*)- and (*E*)-hexen-2-ol (Supplementary Table S3) and lower TA in CV samples (Supplementary Figure S1 and Table S1), together with higher quantities of bound 3-oxo-α-ionol, explained the separation of CV from group 2 along F3 (Figure 1B,D). Mean concentrations of 3-oxo-α-ionol, arising in the berry from the corresponding carotenoid as a function of the amount of light received after véraison [36], were significantly higher in CV and lowest in ADL, LC and MV. Concentrations were correlated to the average amount of light received by these regions (r = 0.50, *p* < 0.001), where CV had the highest level of irradiation (25.1 MJ/m^2^, Supplementary Table S6) in February (i.e., commercial harvest month for all regions except RVL).

Although not observable in the MFA plots, it was possible to discriminate RVL samples from all others in 2015 based solely on the composition of elements. RVL had the lowest Mg content of all samples (Supplementary Table S2, 73 mg/L) and the highest concentrations of Al (2.0 mg/L) and P (175 mg/L).

Examination of the GDD values, minimum and maximum temperatures, and days above 25 °C and 30 °C (Supplementary Table S6) for both vintages and for each region revealed that 2016 was a warmer year than 2015. However, due to rain events during early February 2016 (i.e., harvest period, Supplementary Table S6), the harvest date for all sites except RVL was not more advanced in relation to the previous year (Supplementary Table S1). In 2016, the first three dimensions of the MFA plot (Figure 2) accounted for 56% of the total variance. Discrimination along F1 was again driven in the positive direction by Cu, Zn, benzyl alcohol, and 2-phenylethanol concentrations. Separation along F2 was related to TA in the positive direction and total soluble solids (TSS, as °Brix) in the negative (Figure 2C), and F3 was mainly driven in the positive direction by the presence of higher amounts of (*Z*)-3-hexen-1-ol and to a lesser extent Mg, and in the negative direction by TSS (Figure 2D).

Discrimination of ADL was observed mainly along F1 (Figure 2B, F1/F3, lower right quadrant) and driven by its higher concentrations of free 2-phenylethanol and benzyl alcohol (Supplementary Table S3, Supplementary Figure S1), Cu and Zn content (Supplementary Table S2), and lower 2-ethyl-1-hexanol concentration. This confirms the trends observed during 2015, and matches with the previous results for this region [37]. Given that 2016 was a warmer year, the importance of cooler nights to the decrease in concentration of 2-phenylethanol and benzyl alcohol seems to have been accentuated. Strong negative correlations were found for the 2016 vintage between night time temperature and the respective concentrations of these alcohols (r = −0.78 and r = −0.71, *p* < 0.0001). Unlike 2015, RVL samples in 2016 could be easily distinguished from all other regions in the MFA multivariate space along F2, mainly because of their higher concentrations of TA (Figure 2C and Supplementary Table S1, 9.5 g/L as tartaric acid) and Ca (65 mg/L), and lower pH.

Pearson correlation analysis showed that for both years, the presence of 2-ethyl-1-hexanol was favoured by higher GDD values (r_2015_ = 0.53 and r_2016_ = 0.64, *p* < 0.0005). BV and CV, which had the highest mean January and February maximum temperatures after RVL (Supplementary Table S6), were observed to the extreme left of F1 (Figure 2A) opposite ADL, due to their lower concentrations of 2-phenylethanol and benzyl alcohol, as well as higher amounts of linalool, 2-ethyl-1-hexanol, ethyl octanoate, Ca, and B (Figure 2C). CV could be discriminated from BV along F3 (Figure 2B) mainly due to a slightly higher concentration of (*Z*)-3-hexen-1-ol and lower TSS (Figure 2D). LC and EV exhibited an intermediate profile in 2016 between the warmer and cooler regions, probably due to warmer nights compared to 2015.

#### 2.1.3. Major and Trace Elements

Although most elements did not show any marked trends between regions across both vintages, some patterns could be observed for certain elements such as Zn and P. For both vintages, the mean concentrations of Zn in ADL were significantly different and higher than those of all other regions according to Tukey’s HSD post hoc test, followed by EV, and those from BV were significantly lower than those from all other regions (Supplementary Table S2). This can be partly attributed to the application of seaweed extract (*Ascophyllum nodosum*) to vines in the ADL vineyards, which has been shown to significantly increase vine Zn content [38]. Perusal of the Australian Soil Resource Information System (ASRIS) [39] also showed a higher concentration of this element in the root active zone in the soils of the ADL region than in all other regions (with the exception of RVL for which such information was not available). Zn plays an essential structural and functional role in yeast cells and is required as an essential cofactor for enzymatic activity where it binds to catalytic active sites and acts as an activator of the terminal alcohologenic Zn-metalloenzyme ethanol dehydrogenase during fermentation. Zn deficiencies lead to slow or incomplete fermentations [40]. Comparison of means showed that P concentrations were significantly different and higher in the RVL during both vintages (and MV in 2015), and lowest in ADL and EV. The corresponding ASRIS data sheets [39] indicated that P contents were marginal in the selected ADL and EV sites. Additionally, unlike RVL, both the ADL and EV sites had soils with an acidic pH, which lowers the availability to the plant of any P present due to fixation by aluminium or iron [41]. Higher levels of Cu were measured in ADL and EV samples, with the lowest mean contents being found in CV (Supplementary Table S2, Supplementary Figure S1), which reflect the composition of these regions’ soils. The mean relative concentrations of Fe were significantly different and higher for both years in ADL than in all other sites (Supplementary Figure S1). Fe and Cu are needed at low concentrations as co-factors in cell metabolism, however, they can be toxic to yeast development when present at around 6 mg/L or higher [42]. Fe and Cu concentrations were well below this value for all samples in both years. Berry concentrations of Mg changed from year to year for most regions, but EV had the highest mean relative concentrations of this element in both vintages. Mg is an essential mineral for good fermentation performance–it is involved in every phosphate-transferring enzymatic process–and is crucial to metabolic activities including glycolysis and alcoholic fermentation [43]. According to Sommer et al. [44], *S. cerevisiae* preferentially utilises Mg^2+^ cations during fermentation and biomass formation.

#### 2.1.4. Amino Acids

Overall, the amino acid results showed no discernible pattern contributing to the separation of samples by region in either year: any regional effects that may have existed are seemingly overshadowed by other factors. Amino acid concentrations can be greatly impacted by the degree of berry maturity and water stress, level of fertilisation, and other viticultural parameters [45], and considerable variation between vintages has been reported previously [46]. Amino acids are important contributors to quality as they act as precursors to many key compounds including, but not restricted to, higher alcohols, aldehydes, and esters [47]. The Ehrlich pathway gives rise to higher alcohols through the degradation of the corresponding amino acid [48]. Higher alcohols can have both positive and negative effects on wine quality depending on their concentrations [47,48] and can be transformed during fermentation into their corresponding fruity esters. Esters are crucial to Chardonnay wine quality, as they impart desirable aromas and constitute one of the main odorant classes of this grape variety [49]. Amino acids also affect yeast metabolism, regulating the formation of compounds that are detrimental to wine quality such as the volatile sulfur compounds (e.g. hydrogen sulfide, methyl mercaptan) [47]. Looking at the data more closely, RVL samples contained higher levels of aspartic acid in 2016 (Supplementary Table S4, 84 mg/L) that made them distinct from all other regions. Likewise, ADL could be described by higher contents of alanine (249 mg/L), serine (127 mg/L), glutamic acid (183 mg/L) and glutamine + glycine (GLN + GLY, 246 mg/L) than all other regions for that vintage. EV had the second highest levels of serine (120 mg/L) and glutamic acid (161 mg/L) in 2016. Relative contents of phenylalanine were lowest in ADL, BV, and RVL in both years.

Consistent with the work of Stines et al. [50], proline and arginine were the two predominant amino acids in the grapes (in both vintages) and the content of proline was in line with the average of 742 mg/L published by Amerine and Ough [51]. Unlike other grape varieties, Chardonnay favours proline accumulation from véraison onwards, which can be potentially problematic. Proline is non-assimilable by yeast and higher amounts relative to forms of yeast assimilable nitrogen (YAN) can lead to challenging fermentations due to the lower total amount of YAN available. As a whole, higher amounts of proline were observed during 2015 than 2016. During both years, lysine, isoleucine, leucine, tyrosine and β-alanine were found at the lowest concentrations. Their combined concentrations amounted to less than 100 mg/L or 5% of the total amino acid content. Isoleucine, leucine, and lysine have also been cited by Hernández-Orte et al. [46] as being minor amino acids in Tempranillo grapes.

#### 2.1.5. Glycosides

Similarly to amino acids, free and bound volatiles did not differ specifically according to the geographical origin of the grapes. The profile of bound volatiles in the form of glycosidic aroma precursors is dependent on maturity level and weather as well as grapevine canopy [19]. Consequently, the results obtained for bound volatiles for each year and region varied considerably. For 2015 samples, in contrast to what was observed for the corresponding free alcohols with respect to night time temperature, hydrolytically-released 2-phenylethanol and benzyl alcohol concentrations were highest in MV and LC samples (Figure 1, Supplementary Table S5), with these being two of the regions with the highest night time temperatures. MV also had significantly higher levels of TDN, α-terpineol, and 4-vinylguaiacol (4VG) than all other regions. Formation of C_13_-norisoprenoids, including TDN, is affected by sunlight and heat. TDN is a carotenoid degradation product, and the concentrations of its carotenoid precursors (lutein and β-carotenoid) have been shown to increase in grapes from hot regions [52,53]. Likewise, the formation of glycosidically-bound monoterpenoids such as α-terpineol is influenced by berry microclimate, where levels are higher in fruit with greater sunlight exposure (and higher bunch temperature) [54]. ADL together with CV presented the highest levels of 2,6-dimethyl-7-octene-2,6-diol and MV had the lowest.

In 2016, there were no significant differences between the concentrations of hydrolytically-released 2-phenylethanol and benzyl alcohol in samples from the different regions (Supplementary Table S5). This seems to be partly explained by the overall higher temperatures in 2016. BV and CV had the highest mean concentrations of vitispirane, 2,6-dimethyl-7-octene-2,6-diol, 5-methylfurfural (5-MF), β-damascenone, α-terpineol, and β-ionone. Higher levels of 5-MF, vitispirane, β-ionone and β-damascenone accorded with preceding results for BV and CV, which were compared to ADL and EV in that study [37]. As observed previously, the presence of these four compounds in the berries exhibited significant correlations to GDD (r = 0.53, r = 0.56, r = 0.56, and r = 0.43 for 5-MF, vitispirane, β-ionone, and β-damascenone, respectively, *p* < 0.001), as well as to February maximum temperatures and number of days over 25 °C during January and February (Supplementary Table S6). As stated previously, carotenoid degradation increases in hotter climates with the corresponding augmentation of C_13_-norisoprenoid concentrations in the berry [52,53]. Additionally, heat stress in berries during ripening results in a partial anaerobic metabolism and the production of ethanol, CO_2_, and fermentation by-products including 5-MF [55,56,57]. Correspondingly, the mean concentrations of α-terpineol and 5-MF were lowest in EV and LC. These two regions had less days over 25 °C during January and February in 2016 than all other sites, which appears to also have had an impact on the production of 3-methyl-1-butanol, (*E*)- and (*Z*)-linalool oxide, linalool, (*E*)-2-hexenal, and 4VG, which were lower in these samples. 3-Oxo-α-ionol was again found in the highest concentrations in CV, which had the highest solar exposure of all regions harvested in February (24.8 MJ/m^2^, Supplementary Table S6). Despite RVL being the hottest region from which samples were sourced, most of the abovementioned compounds were found at the lowest concentrations in RVL samples, likely as a result of the earlier harvest dates in 2016. Grapes from this region were less ripe than the remainder (Supplementary Table S1, TSS = 18.9 °Brix, TA = 9.5 g/L) and therefore most C_13_-norisoprenoids and monoterpenoids, which are synthesised during ripening [36], were found in lower concentrations. Methyl vanillate, 2,6-dimethoxyphenol, α-terpinene, hexanoic acid, octanoic acid, 4VG, guaiacol, 3-methyl-1-butanol, (*E*)-2-hexenal, and (*E*)- and (*Z*)-linalool oxides were higher in ADL than all other regions. As a whole, due to the effect of rain during ripening in 2016, lower amounts of bound monoterpenoids and C_13_-norisoprenoids were observed compared to 2015 [58,59].

### 2.2. Prediction of Geographical Indication Based on Composition Variables

**Figure 3:**
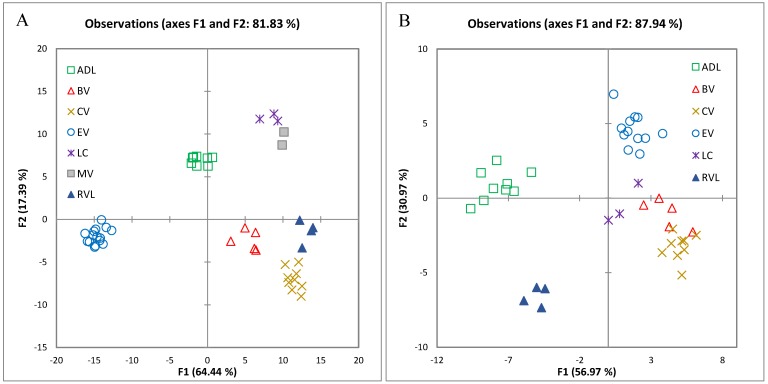

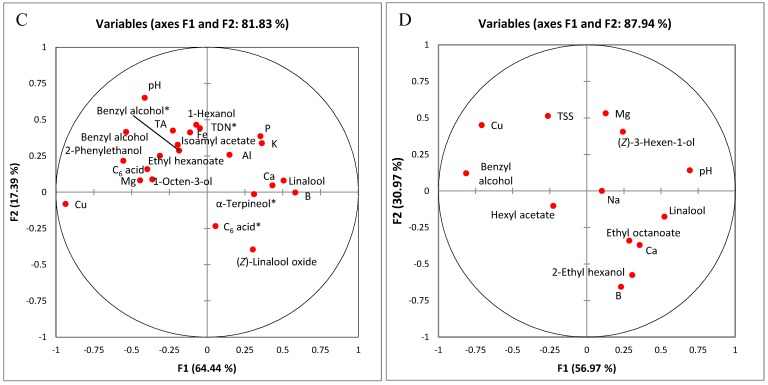
Distribution of grape samples in the two coordinate system defined by discriminant analysis showing the two canonical variables with the highest discrimination power. Scores projected onto F1 and F2 for 2015 (**A**) and 2016 (**B**) show samples grouped according to GI where ADL, Adelaide Hills; BV, Barossa Valley; CV, Clare Valley; EV, Eden Valley; LC, Langhorne Creek; MV, McLaren Vale; RVL, Riverland. Loadings of variables used in the analysis are shown for 2015 (**C**) and for 2016 (**D**) where * denotes volatiles detected after hydrolysis of glycosides extracted from juice. Abbreviations (e.g., TSS, TDN) are the same as specified in the text.

A number of studies have been conducted to discriminate wines according to geographical origin but very few have done so on grapes [4]. Amongst the grape studies, successful results have been obtained when using mineral elements for clearly distinct geological regions [10,11]. However, these studies have failed to distinguish regions that are closer in proximity based exclusively on a single class of berry constituents such as elements. Unlike PCA and MFA, discriminant analysis (DA) is a supervised classification technique that requires prior knowledge of class membership. DA was carried out using backward stepwise selection of variables and full cross-validation to predict the membership of a sample to a particular GI (Figure 3). Overall classification rates for the years 2015 (*n* = 50) and 2016 (*n* = 45) were 100%. Based on the results of MFA, the variables used by the model to discriminate between regions in 2015 were Ca, K, Mg, Na, Fe, B, Cu, P, Al, the free volatiles isoamyl acetate, ethyl hexanoate, hexanoic acid, 1-hexanol, (*E*)-3-hexen-1-ol, (*E*)- and (*Z*)-2-hexen-1-ol, (*Z*)-linalool oxide, 1-octen-3-ol, linalool, 2-phenylethanol, benzyl alcohol, pH, and TA, and the bound volatiles α-terpineol, hexanoic acid, TDN, and benzyl alcohol (Figure 3C). In 2016, classification relied on the presence of Ca, Mg, Na, B, Cu, the free volatiles hexyl acetate, (*E*)-3-hexen-1-ol, ethyl octanoate, 2-ethyl-1-hexanol, linalool, benzyl alcohol, and pH and TSS (Figure 3D). These results indicate the potential for the classification of grapes according to origin but should be considered as exploratory; a larger set of samples from each GI is required to confirm these outcomes.

### 2.3. Correlating Grape Grading and Grape Composition

**Figure 4:**
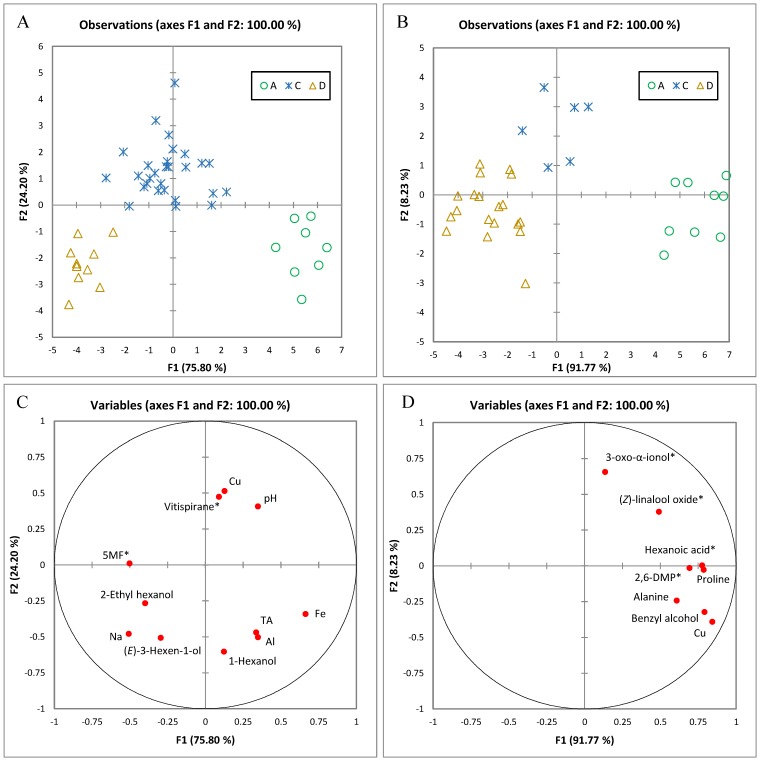
Distribution of grape samples in the two coordinate system defined by discriminant analysis showing the two canonical variables with the highest discrimination power. Scores projected onto F1 and F2 for 2015 (**A**) and 2016 (**B**) shows samples grouped according to quality grade (A, C and D). Loadings of variables used in the analysis are shown for 2015 (**C**) and for 2016 (**D**) where * denotes volatiles detected after hydrolysis of glycosides extracted from juice. Abbreviations (e.g., TA, 5-MF, 2,6-DMP) are the same as specified in the text.

Grades are normally allocated to Chardonnay grapes by winemakers in Australia (and likely elsewhere) based on the location and previous knowledge of the vineyard, basic chemical parameters such as pH, TA and TSS, and flavour profile as judged during berry tasting in the vineyard [60]. However, the tasting process does not necessarily take into account the true flavour composition and aroma potential of the berries. Key volatile compounds such as the monoterpenoids and C_13_-norisoprenoids are synthesised through biological pathways that are independent and, depending on climatic and viticultural conditions, often asynchronous to sugar accumulation [61]. Conscious that technological maturity (based on basic chemical parameters to determine harvest timing) and aroma maturity do not always coincide, companies are looking to develop indices which include volatile compounds to better stream grapes into the different desired fruit categories [62]. MFA of significantly different attributes (according to allocated grade) was carried out on berry composition for 2015 and 2016, and followed up with DA with full cross-validation to identify the parameters that most influenced the allocated grades (Figure 4). In 2015 and 2016, 100% of the samples were correctly classified as A-, C- or D-grade. These correct classification rates exceed the grade prediction results for Chardonnay grapes mentioned by Smith [63]. However, as a result of the large effect that vintage year has on the composition of the berries, the different grading systems used by winemakers in each study, and the different measurements taken into account, some variances could be observed between the results of the prediction models from our work versus those reported by Smith. Classification in 2015 was based on the concentration of Na, Fe, Cu, Al, C_6_ alcohols (1-hexanol, (*E*)-3-hexen-1-ol, 2-ethyl-1-hexanol), pH, TA, and hydrolytically-released vitispirane and 5-MF. Samples corresponding to A-grade fruit had higher TA, higher concentrations of Al and Fe, and lower levels of bound 5-MF. C-grade samples had overall lower concentrations of 1-hexanol and Al, and higher concentrations of Cu and vitispirane, whereas D-grade samples were characterised by higher levels of (*E*)-3-hexen-1-ol, 2-ethyl-1-hexanol, Na, and bound 5-MF, and lower pH and lower concentrations of Cu and vitispirane. Classification in 2016 depended on the levels of Cu, benzyl alcohol, alanine, proline, and hydrolytically-released (*Z*)-linalool oxide, hexanoic acid, 3-oxo-α-ionol, and 2,6-dimethoxyphenol (2,6-DMP). A-grade samples were correctly classified based mainly on their higher mean concentrations of benzyl alcohol, Cu, proline, alanine, and bound hexanoic acid and 2,6-DMP. C-grade samples had higher levels of bound 3-oxo-α-ionol, and D-grade samples had the lowest levels of hydrolytically-released 3-oxo-α-ionol and (*Z*)-linalool oxide.

#### Impact of Climate on Berry Quality Grades

Climate has been shown to have a significant impact on the final composition and quality of both grapes and wines [3]. Pearson correlation analysis revealed a significant negative correlation between allocated Chardonnay grape quality grade and GDD (r_2015_ = −0.80 and r_2016_ = −0.60, *p* < 0.0005). RVL had the highest GDD for both years (2370 and 2670, respectively, Supplementary Table S6), and the corresponding grapes were graded as D, in line with the correlation analysis results. However, whereas RVL grapes constituted the lowest grade assigned in 2015, grapes from certain areas in CV and EV, and all BV vineyards, were classified as D in 2016, making this relationship with GDD less clear. Temperature has a large effect on grape composition by impacting the rate of photosynthesis and the formation and degradation of important metabolites during maturation, and thus affects quality [3]. Higher temperatures decrease the concentration of organic acids, namely malate [64], and increase the amount of shrivelled and sunburnt berries [65]. This is especially relevant to Chardonnay grapes, which are very susceptible to sunburn under high temperature conditions. Sunburn increases the concentration of secondary phenolic compounds, and as shown for Sauvignon blanc grapes, shifts wine aroma profiles from fresh and fruity to phenolic and neutral, with a consequent loss of quality [66,67]. Closer examination of our results revealed that the effect of GDD on allocated grade was partly driven by the negative influence of higher night time temperatures (measured as minimum average temperature), mainly in January (r_2015_ = −0.78 and r_2016_ = −0.85, *p* < 0.005) but also in February (r_2015_ = −0.70, *p* < 0.001), and maximum average temperatures in February (r_2015_ = −0.78 and r_2016_ = −0.59, *p* < 0.001). A significant negative effect of temperatures above 30 °C was observed on the quality of the grapes, with r_2015_ = −0.87 and r_2016_ = −0.70 (*p* < 0.0001). This means that higher temperatures, and particularly higher night time temperatures, negatively affected the development of positive characteristics in Chardonnay grapes. Higher temperatures cause volatilisation of aroma compounds such as the monoterpenes and enhance their biotransformation and degradation, and decrease benzenoid concentrations [68]. According to temperature sensitivity models, rises in temperature result in significant losses of quality for Chardonnay grapes (measured as the price paid per tonne of grapes) [69]. Significant correlations between the allocated grade and precipitation that occurred during January and February were found only for the 2016 vintage (r_Jan_ = 0.53 and r_Feb_ = 0.34, *p* < 0.05). Precipitation during both months was moderate, and distributed over a period of several days, which gave viticulturists enough time to apply fungicides (when possible) and adjust irrigation regimes to avoid the potential for berry burst and increased disease pressure. The moderate levels of rain in 2016 seem to have delayed maturity slightly, giving berries the possibility to ripen more slowly despite the warmer weather.

## 3. Materials and Methods

### 3.1. Chemicals

All chemicals were of analytical reagent grade unless otherwise stated. Water was obtained from a Milli-Q purification system (Millipore, North Ryde, NSW, Australia). All aroma reference compounds were obtained from either Sigma-Aldrich (Castle-Hill, NSW, Australia), Alfa-Aesar (Ward Hill, MA, USA), Riedel-de Haёn (Seelze, Germany), or Hopkin and Williams (London, UK). Deuterium-labelled internal standards consisting of d_4_-3-methyl-1-butanol, d_3_-hexyl acetate, d_13_-1-hexanol, d_5_-phenyl ethanol, and d_19_-decanoic acid were supplied by CDN Isotopes (Point-Claire, Quebec, CN, Canada), and d_5_-ethyl dodecanoate was synthesised previously [70].

### 3.2. Grapes

*V. vinifera* L. cv. Chardonnay berry bunch samples (4 kg) were randomly harvested from commercial vineyards in the Adelaide Hills (*n* = 8), Eden Valley (n_2015_ = 15, n_2016_ = 12), Clare Valley (*n* = 9), Barossa Valley (*n* = 5), Langhorne Creek (*n* = 3), McLaren Vale (only 2015, *n* = 2), and the Riverland (*n* = 4), South Australia, at commercial maturity (~21.6 °Brix) during the 2015 and 2016 vintages. The number of samples collected was the same for each site for both vintages unless stated otherwise. Regions were located around the following GPS coordinates: Adelaide Hills, S: 34°56′15 and E: 138°52′36; Eden Valley, S: 34°37′03 and E: 139°02′27; Clare Valley, S: 33°57′16 and E: 138°39′12; Barossa Valley, S: 34°38′26 and E: 138°53′41; Langhorne Creek, S: 35°19′42 and E: 138°58′ 35; McLaren Vale, S: 35°11′12 and E: 138°33′24; Riverland, S: 34°04′28 and E: 139°52′03. Samples were transported on ice and were carefully stored at −20 °C until required for analysis and were destemmed as necessary while frozen. Quality grades (scale of A–E, where A was the highest grade) provided by the wine companies were allocated to fruit from each vineyard based on wine sensory characteristics after the wines had been finalised.

### 3.3. Juice Basic Chemical Analysis

Titrable acidity (TA, expressed as g/L of tartaric acid at pH 8.2) and pH were measured using a combined pH meter and autotitrator (CompacTitrator, Crison Instruments, S.A., Allela, Spain) [71]. Total soluble solids of grapes (TSS, expressed as °Brix) was determined using a digital refractometer (Atago pocket, Atago Co., Ltd., Tokyo, Japan).

### 3.4. Headspace-Solid Phase Microextraction-Gas Chromatography-Mass Spectrometry (HS-SPME-GC-MS) of Free Volatiles from Grapes

A total of 28 compounds were semi-quantified in Chardonnay juice as previously described [37]. All analyses were performed in duplicate.

### 3.5. Analysis of Hydrolysed Grape Glycosides by Gas Chromatography-Mass Spectrometry (GC-MS)

Glycosidic precursors in grapes were measured using the method described by Hernandez-Orte et al. [19] and the GC-MS instrumentation described previously [37].

### 3.6. Element Analysis by Inductively Couple Plasma-Optical Emission Spectroscopy (ICP-OES)

Juice samples were analysed for aluminium (Al), arsenic (As), boron (B), cadmium (Cd), calcium (Ca), cobalt (Co), chromium (Cr), copper (Cu), iron (Fe), lead (Pb), magnesium (Mg), manganese (Mn), molybdenum (Mo), nickel (Ni), potassium (K), phosphorus (P), selenium (Se), sodium (Na), sulfur (S), and zinc (Zn) by ICP-OES after acid digestion. Analyses were performed by the CSIRO Analytical Services Unit according to Wheal et al. [72].

### 3.7. Amino Acid Analysis by HPLC with Diode Array Detection

Amino acids in grape samples were quantified according to Boss et al. [70] using an AccQ Fluor reagent kit (Waters Corporation, Milford, MA, USA) and solid-phase extraction clean-up step. The glycine peak co-eluted with glutamine and was therefore reported together as “GLN + GLY”.

### 3.8. Climatic and Soil Data

Weather data (maximum and minimum daily temperatures (°C), daily rainfall (mm), and solar exposure (MJ/m^2^)) were obtained from the Australian Bureau of Meteorology [73]. Growing degree days (GDD base 10 °C) were calculated for the active vegetation period (1 October–30 April). Soil profiles were obtained from the Australian Soil Resource Information System based on proximity and GPS coordinates [39].

### 3.9. Data Analysis

XLSTAT (version 2014.05.03, Addinsoft, Paris, France) was used to conduct two-way analysis of variance (ANOVA) on all instrumental measurements to test the effect of vintage and region. Principal component analysis (PCA) was conducted on the means of all significantly different parameters (region as the explaining variable) after normalisation (1/standard deviation) to elucidate the differences between grapes according to the vintage. The number of principal components (PC) was selected based on their eigenvalues and scree plots [74]. Multiple factor analysis (MFA) was conducted using XLSTAT on significantly different compositional variables (according to region and quality grade). RV cut-off values for inclusion were set at 0.6 [75]. Discriminant analysis (DA) was then performed to classify samples into their respective regions of origin and quality grade. The analysis was performed with XLSTAT on the variables selected by MFA analysis and using stepwise selection of variables according to their discriminating power, as measured by an *F* statistic. Performance of all classifications was evaluated by leave-one out full-cross validation.

## 4. Conclusions

In accord with other reports, the year of vintage exerted a strong influence on the composition of Chardonnay grapes originating from seven different GI in South Australia, as a result of changes in the weather between 2015 and 2016. Measurement of elements (minerals), amino acids, basic chemical composition, and free and bound volatile compounds, in conjunction with chemometric treatment of the data sets, allowed the discrimination of the different regions using both MFA and DA. Two of the main compounds driving the separation in both years were 2-phenylethanol and benzyl alcohol, the concentrations of which were higher in the cooler sites. These results indicated that preservation of 2-phenylethanol and benzyl alcohol is highly correlated to cooler nights rather than cooler overall mean temperatures.

Mineral composition proved to be a useful parameter to discriminate between grapes from different origins, which agrees well with other studies. Concentrations of Zn, Cu, and Mg contributed to the separation of ADL and EV from other GI in both vintages, indicating possible markers related to origin that can be used consistently, and independent of weather and vintage. ADL berries also contained high levels of Zn, Fe, and Al that were characteristic of the soil in the ADL region. Relationships between soil and berry mineral composition could be further corroborated with renewed soil analysis of each particular site, as soil composition can be modified through viticultural practices.

With respect to bound volatiles, the concentration of 3-oxo-α-ionol was highest in CV during both vintages, consistent with this region having one of the highest levels of solar radiation. Based on the first three factors of the MFA, successful classification models for GI were built using DA. All samples were correctly classified according to their region of origin in 2015 and 2016. Successful classification models were also built according to the allocated grape quality grade. Correct grade classification of 100% of the samples in 2015 was based on the concentrations of Cu, Na, Fe, Al, 1-hexanol, (*E*)-3-hexen-1-ol, 2-ethyl-1-hexanol, pH, TA and hydrolytically-released vitispirane, and 5-MF. In 2016, 100% of the samples were correctly classified based on the levels of Cu, benzyl alcohol, alanine, proline, and hydrolytically-released (*Z*)-linalool oxide, 3-oxo-α-ionol, 2,6-DMP, 5-MF, and hexanoic acid. However, these results need to be confirmed with further studies encompassing a larger number of samples, especially for some regions.

Pearson correlation revealed the effect of climate on the Chardonnay berry quality grades, with significant negative correlations found between GDD and quality grade for both years, and a particularly large negative influence of higher night time temperatures on allocated grape quality. These results provide some insight into the factors to target in order to manipulate fruit quality in warm regions, particularly with respect to diurnal temperature differences. Thus, methods of bunch-zone cooling could be investigated, especially during heatwave events, or more optimal vineyard locations chosen in view of changes to the climate that are envisaged to occur over the coming decades.

## Supplementary Materials

The following are available online at http://www.mdpi.com/1420-3049/22/2/218/s1, Figure S1. Boxplots of main compositional variables used to discriminate between Chardonnay grape samples from seven GI in South Australia and quality grades in 2015 and 2016: (A) Fe 2015, (B) Fe 2016, (C) Cu 2015, (D) Cu 2016, (E) Zn 2015, (F) Zn 2016, (G) Al 2015, (H) Al 2016, (I) 2-Phenylethanol 2015, (J) 2-Phenylethanol 2016, (K) Benzyl alcohol 2015, (L) Benzyl alcohol 2016, (M) Total soluble solids 2015, (N) Total soluble solids 2016, (O) Titratable acidity 2015, (P) Titratable acidity 2016, (Q) β-Ionone* 2015, (R) β-Ionone* 2016, (S) 5-MF* 2015, (T) 5-MF* 2016; Table S1. Harvest date and mean values of pH, total soluble solids (°Brix), and titratable acidity (TA) for Chardonnay grapes harvested from seven GI in South Australia in 2015 and 2016; Table S2. Mean element concentrations (mg/L) in harvest samples of Chardonnay berries collected from seven GI within South Australia in 2015 and 2016; Table S3. Mean concentrations (expressed as mg/L of deuterated internal standard) of free volatile compounds determined in harvest samples of Chardonnay berries collected from seven GI within South Australia in 2015 and 2016; Table S4. Mean content (mg/L) of amino acids in Chardonnay berries at harvest from seven GI in South Australia in 2015 and 2016; Table S5. Mean concentrations of hydrolytically-released volatile compounds determined in harvest samples of Chardonnay berries collected from seven GI within South Australia in 2015 and 2016; Table S6. Weather data for all regions sampled, including mean, minimum, maximum, and highest temperature for the months of January and February, number of days when the temperature exceeded 25 and 30 °C during the January-February period, GDD values, and total rainfall and solar exposure for the months of January and February.
