# Supplementary material for: Exploring the Effects of Geographical Origin on the Chemical Composition and Quality Grading of Vitis vinifera L. cv. Chardonnay Grapes
Source: Molecules. 2017 Jan 31;22(2):218. doi: 10.3390/molecules22020218 (PMC6155627; doi:10.3390/molecules22020218)
Supplement: Supplementary file 1 [file molecules-22-00218-s001.pdf]

Supplementary Information: Exploring the Effects of Geographical Origin on the Chemical Composition and Quality Grading of *Vitis vinifera* L. cv. Chardonnay Grapes

Joanna M. Gambetta [1], Daniel Cozzolino [2], Susan E.P. Bastian [1] and David W. Jeffery [1,*]

Table of Contents

Figure S1. Boxplots of main compositional variables used to discriminate between Chardonnay grape samples S2
Table S1. Harvest date and mean values of pH, total soluble solids (°Brix) and titratable acidity (TA) for Chardonnay grapes S4
Table S2. Mean element concentrations in harvest samples of Chardonnay berries S5
Table S3. Mean concentrations of free volatile compounds determined in harvest samples of Chardonnay berries S6
Table S4. Mean content of amino acids in Chardonnay berries at harvest S9
Table S5. Mean concentrations of hydrolytically-released volatile compounds determined in harvest samples of Chardonnay berries S10
Table S6. Weather data for all regions sampled S13

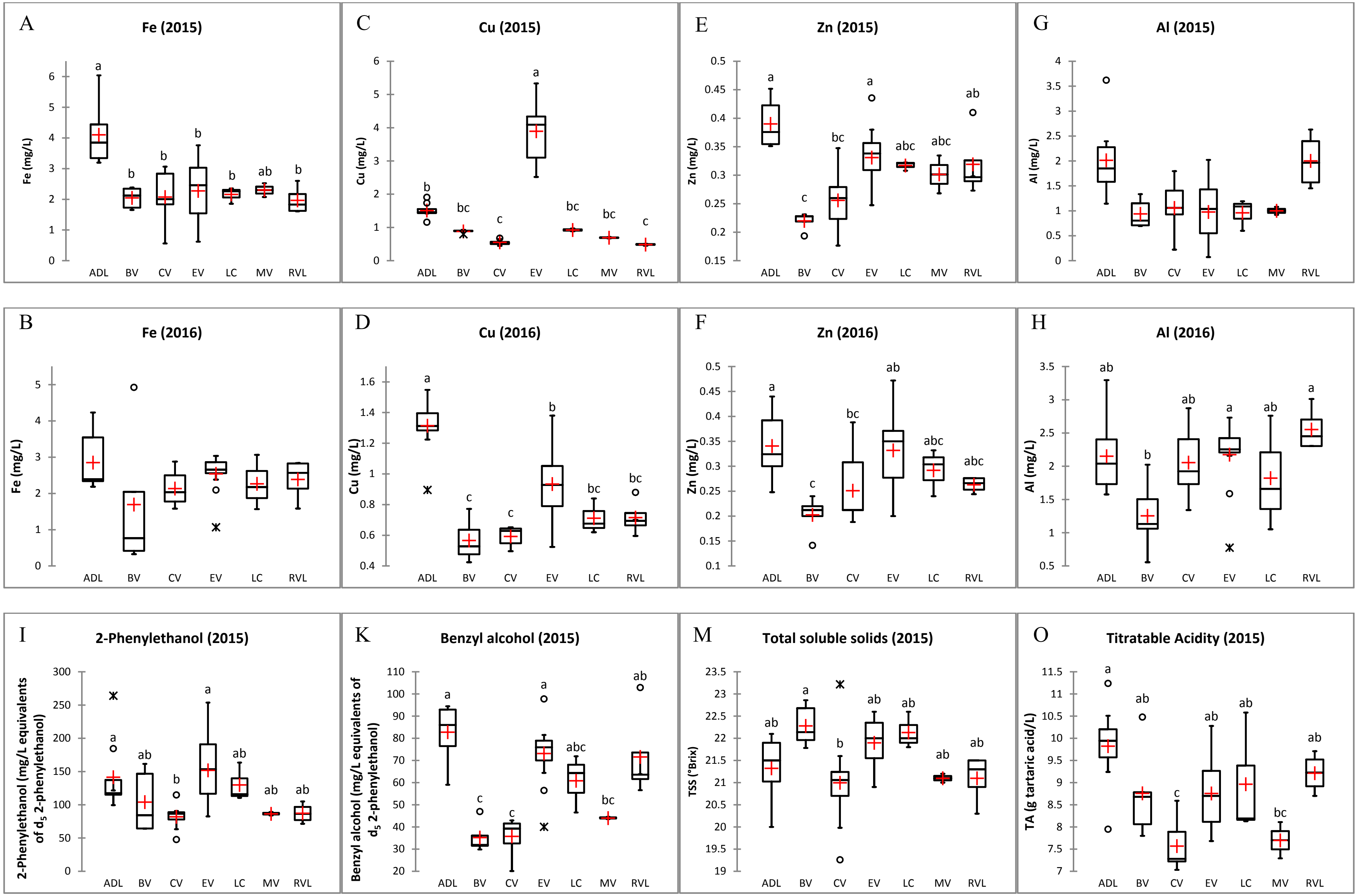
A
Fe (2015)
Fe (mg/L)
ADL BV CV EV LC MV RVL
C
Cu (2015)
Cu (mg/L)
ADL BV CV EV LC MV RVL
E
Zn (2015)
Zn (mg/L)
ADL BV CV EV LC MV RVL
G
Al (2015)
Al (mg/L)
ADL BV CV EV LC MV RVL
B
Fe (2016)
Fe (mg/L)
ADL BV CV EV LC RVL
D
Cu (2016)
Cu (mg/L)
ADL BV CV EV LC RVL
F
Zn (2016)
Zn (mg/L)
ADL BV CV EV LC RVL
H
Al (2016)
Al (mg/L)
ADL BV CV EV LC RVL
I
2-Phenylethanol (2015)
2-Phenylethanol (mg/L equivalents of d5 2-phenylethanol)
ADL BV CV EV LC MV RVL
K
Benzyl alcohol (2015)
Benzyl alcohol (mg/L equivalents of d5 2-phenylethanol)
ADL BV CV EV LC MV RVL
M
Total soluble solids (2015)
TSS (°Brix)
ADL BV CV EV LC MV RVL
O
Titratable Acidity (2015)
TA (g tartaric acid/L)
ADL BV CV EV LC MV RVL

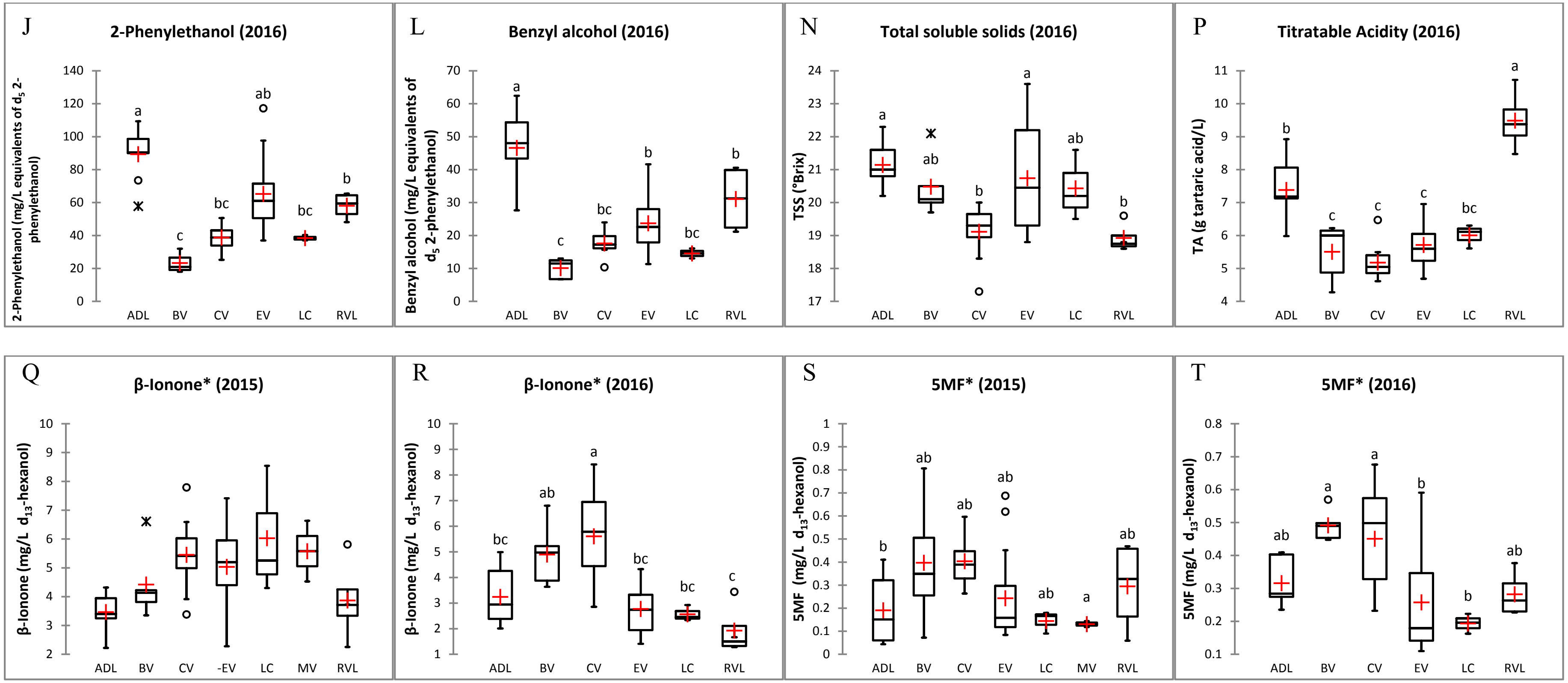

Figure S1. Boxplots of main compositional variables used to discriminate between Chardonnay grape samples from seven GI in South Australia and quality grades in 2015 and 2016: (**A**) Fe 2015, (**B**) Fe 2016, (**C**) Cu 2015, (**D**) Cu 2016, (**E**) Zn 2015, (**F**) Zn 2016, (**G**) Al 2015, (**H**) Al 2016, (**I**) 2-Phenylethanol 2015, (**J**) 2-Phenylethanol 2016, (**K**) Benzyl alcohol 2015, (**L**) Benzyl alcohol 2016, (**M**) Total soluble solids 2015, (**N**) Total soluble solids 2016, (**O**) Titratable acidity 2015, (**P**) Titratable acidity 2016, (**Q**) β-Ionone* 2015, (**R**) β-Ionone* 2016, (**S**) 5MF* 2015, (**T**) 5MF* 2016. ADL, Adelaide Hills; BV, Barossa Valley; CV, Clare Valley; EV, Eden Valley; LC, Langhorne Creek; MV, McLaren Vale (only 2015); RVL, Riverland. * denotes volatiles detected after hydrolysis of glycosides extracted from juice. 5MF, 5-methylfurfural. [a] For each compound, regions with different letters (a, b, c) above the box have significantly different ($p < 0.05$) mean values according to Tukey's (HSD) pairwise comparison

Table S1. Harvest date and mean values of pH, total soluble solids (TSS) and titratable acidity (TA) for Chardonnay grapes harvested from seven Geographical Indications in South Australia in 2015 and 2016. [a]

	**Harvest**		**pH**				**TA(g Tartaric Acid/L)**				**TSS (°Brix)**			
	**2015**	**2016**	**2015**		**2016**		**2015**		**2016**		**2015**		**2016**	
ADL [b]	16/2	16/2	3.5	(0.1)b	3.2	(0.1)b	9.8	(1.0)a	7.4	(0.9)b	21.3	(0.8)ab	21.1	(0.7)a
BV	3/2	2/2	3.2	(0.0)c	3.4	(0.1)a	8.8	(0.9)ab	5.5	(0.8)c	22.3	(0.4)a	20.5	(0.8)ab
CV	30/1	4/2	3.3	(0.1)c	3.4	(0.1)a	7.6	(0.5)c	5.2	(0.5)c	21.0	(1.0)b	19.1	(0.8)b
EV	17/2	18/2	3.5	(0.1)b	3.4	(0.1)a	8.8	(0.8)ab	5.7	(0.6)c	21.9	(0.5)ab	20.7	(1.6)a
LC	10/2	15/2	3.8	(0.1)a	3.4	(0.0)a	9.0	(1.4)ab	6.0	(0.3)bc	22.1	(0.4)ab	20.4	(0.9)ab
MV	6/2	- [c]	3.6	(0.1)ab	-	-	7.7	(0.6)bc	-	-	21.1	(0.1)ab	-	-
RVL	23/1	14/1	3.3	(0.0)c	3.1	(0.0)b	9.2	(0.4)ab	9.5	(0.8)a	21.1	(0.5)ab	18.9	(0.4)b

[a] For each region, means ± SD (duplicate measurements for each sample) with different letters within a column (a, b, c) are significantly different ($p < 0.05$) according to Tukey's (HSD) pairwise comparison. [b] Adelaide Hills (ADL, $n = 8$), Barossa Valley (BV, $n = 5$), Clare Valley (CV, $n = 9$), Eden Valley (EV, $n_{2015} = 15$ and $n_{2016} = 12$), Langhorne Creek (LC, $n = 3$), Riverland (RVL, $n = 4$), McLaren Vale (MV, $n = 2$). [c] -, not sampled.

Table S2. Mean element concentrations (mg/L) in harvest samples of Chardonnay berries collected from seven Geographical Indications within South Australia in 2015 and 2016. [a]

**2015**	**ADL** [b]		**BV**		**CV**		**EV**		**LC**		**RVL**		**MV**	
Calcium	66	(18)	66	(5.7)	74	(9.8)	55	(12)	74	(12)	63	(12)	77	(1.5)
Potassium	1176	(86)ab	1436	(119)a	1168	(239)ab	1010	(230)b	1503	(60)a	947	(57)b	1525	(50)a
Magnesium	97	(10)a	89	(3.7)ab	91	(12)a	96	(6.5)a	92	(3.1)a	73	(1.9)b	85	(2.8)ab
Sodium	16	(3.5)b	40	(14)a	17	(5.4)b	13	(3.4)b	45	(14)a	56	(32)a	41	(9)a
Sulfur	67	(6.6)a	58	(8.9)ab	49	(7.4)b	59	(6.0)ab	53	(2.5)b	60	(4.2)ab	59	(1.2)ab
Iron	4.1	(1.0)a	2.0	(0.3)b	2.1	(0.9)b	2.3	(0.9)b	2.2	(0.3)b	2.0	(0.5)b	2.3	(0.3)ab
Manganese	0.43	(0.08)	0.37	(0.10)	0.52	(0.24)	0.44	(0.17)	0.33	(0.04)	0.32	(0.07)	0.42	(0.09)
Boron	3.8	(0.51)abc	3.4	(0.4)c	5.2	(1.4)ab	3.6	(0.5)bc	5.3	(0.4)a	5.4	(0.8)a	5.3	(0.4)ab
Copper	1.5	(0.2)b	0.88	(0.05)bc	0.55	(0.07)c	3.9	(0.85)a	0.92	(0.04)bc	0.48	(0.02)c	0.69	(0.03)bc
Zinc	0.39	(0.04)a	0.22	(0.02)c	0.26	(0.05)bc	0.33	(0.05)a	0.32	(0.01)abc	0.32	(0.06)ab	0.30	(0.05)abc
Phosphorus	100	(16)c	116	(14)bc	91	(25)c	101	(26)c	162	(29)ab	175	(7.9)a	176	(10)a
Aluminium	2.0	(0.78)	0.94	(0.29)	1.1	(0.5)	0.98	(0.61)	0.96	(0.32)	2.0	(0.6)	1.0	(0.1)
**2016**														
Calcium	39	(13)b	55	(20)ab	61	(14)a	51	(7.0)ab	43	(16)ab	65	(5.2)a	- [c]	
Potassium	1298	(166)	1457	(256)	1512	(141)	1367	(220)	1548	(167)	1414	(239)	-	
Magnesium	80	(3.9)ab	80	(6.3)ab	78	(16)b	100	(24)a	65	(9.6)b	70	(9.3)b	-	
Sodium	16	(4.0)ab	52	(53)a	8.8	(4.2)b	15	(19)ab	24	(5.4)ab	14	(7.2)ab	-	
Sulfur	55	(4.9)	56	(7.0)	54	(8.1)	58	(13)	50	(2.5)	52	(9.7)	-	
Iron	2.9	(0.8)	1.7	(1.9)	2.1	(0.5)	2.5	(0.5)	2.3	(0.8)	2.4	(0.6)	-	
Manganese	0.24	(0.07)	0.29	(0.14)	0.31	(0.07)	0.41	(0.21)	0.21	(0.07)	0.32	(0.05)	-	
Boron	3.7	(0.4)bc	4.3	(0.8)abc	5.3	(1.3)a	3.3	(0.9)c	5.1	(0.5)ab	5.0	(1.0)ab	-	
Copper	1.3	(0.2)a	0.57	(0.14)c	0.59	(0.06)c	0.93	(0.26)b	0.71	(0.11)bc	0.72	(0.12)bc	-	
Zinc	0.34	(0.07)a	0.20	(0.04)c	0.25	(0.07)bc	0.33	(0.07)ab	0.29	(0.05)abc	0.26	(0.05)abc	-	
Phosphorus	91	(16)b	129	(22)ab	98	(23)b	128	(27)ab	137	(40)ab	145	(36)a	-	
Aluminium	2.2	(0.6)ab	1.3	(0.5)b	2.1	(0.5)ab	2.2	(0.5)a	1.8	(0.9)ab	2.6	(0.3)a	-	

[a] For each region, means ± SD (duplicate measurements for each sample) with different letters within a row (a, b, c) are significantly different ($p < 0.05$) according to Tukey's (HSD) pairwise comparison. [b] Adelaide Hills (ADL, $n = 8$), Barossa Valley (BV, $n = 5$), Clare Valley (CV, $n = 9$), Eden Valley (EV, $n_{2015} = 15$ and $n_{2016} = 12$), Langhorne Creek (LC, $n = 3$), Riverland (RVL, $n = 4$), McLaren Vale (MV, $n = 2$). [c] -, not sampled.

Table S3. Mean concentrations (expressed as mg/L of deuterated internal standard) of free volatile compounds determined in harvest samples of Chardonnay berries collected from seven Geographical Indications within South Australia in 2015 and 2016. [a]

**2015**	**ADL [b]**		**BV**		**CV**		**EV**		**LC**		**RVL**		**MV**	
Ethyl esters														
Ethyl pentanoate* [c]	15	(7.1)b	16	(6.5)b	13	(2.9)b	18	(9)b	63	(54)a	15	(12)b	85	(42)a
Ethyl hexanoate*	3.9	(1.5)	3.3	(1.5)	3.6	(1.0)	7.2	(4.4)	8.7	(4.6)	2.7	(0.6)	11	(4)
Ethyl octanoate*	1.0	(1.3)	0.68	(0.72)	0.32	(0.15)	4.19	(11)	0.90	(0.08)	0.56	(0.51)	1.89	(1.6)
Diethyl succinate**	0.24	(0.27)ab	0.34	(0.29)ab	0.08	(0.03)b	0.43	(0.42)a	0.22	(0.15)ab	0.13	(0.08)ab	0.20	(0.08)ab
Acetate esters														
Isoamyl acetate*	2.5	(2.2)ab	3.7	(4.5)ab	1.3	(0.4)b	6.2	(6.6)ab	12	(6.1)a	2.1	(1.6)ab	12	(0.0)ab
Hexyl acetate*	3.0	(2.3)	3.0	(1.5)	1.3	(0.5)	2.9	(2.3)	2.8	(1.3)	2.1	(1.4)	3.9	(1.9)
Alcohols														
3-Methyl-1-butanol*	7.5	(2.9)c	6.9	(4.1)c	7.7	(1.9)c	13	(7.4)bc	24	(17)ab	5.5	(0.4)c	43	(23)a
1-Hexanol*	219	(49)a	207	(16)ab	132	(14)b	177	(34)ab	181	(8)ab	206	(81)ab	220	(25)a
(*E*)-3-Hexen-1-ol*	0.68	(0.21)ab	1.0	(0.24)a	0.63	(0.10)b	0.59	(0.16)b	0.70	(0.20)ab	0.77	(0.29)ab	1.0	(0.06)a
(*Z*)-3-Hexen-1-ol*	11	(3.1)a	7.0	(0.7)b	8.8	(2.7)ab	8.0	(1.2)ab	7.3	(0.8)ab	7.2	(2.1)ab	5.5	(0.4)b
(*E*)-2-Hexen-1-ol*	66	(30)abc	103	(15)ab	60	(14)abc	44	(20)c	57	(7)bc	83	(44)abc	108	(2)a
(*Z*)-2-Hexen-2-ol*	1.8	(0.6)ab	2.0	(0.4)ab	1.5	(0.3)ab	1.3	(0.8)b	2.3	(0.7)ab	1.9	(0.3)ab	2.8	(0.0)a
1-Octen-3-ol*	16	(3)	14	(6)	9.4	(1)	17	(7)	11	(3)	18	(10)	12	(2)
2-Ethyl-1-hexanol*	2.0	(0.4)b	3.6	(1.1)ab	3.2	(0.4)ab	2.3	(0.5)b	3.6	(1.9)ab	7.0	(7.0)a	4.7	(0.1)ab
1-Octanol*	1.6	(0.75)a	1.0	(0.47)ab	0.92	(0.25)b	1.3	(0.26)ab	1.5	(0.51)ab	1.7	(1.4)a	1.7	(0.5)a
2-Phenylethanol**	142	(56)a	104	(47)ab	82	(19)b	152	(49)a	130	(29)ab	87	(15)ab	87	(4)ab
Benzyl alcohol**	83	(13)a	35	(7)c	36	(8)c	73	(13)a	61	(13)abc	72	(21)ab	44	(1)bc
Isoprenoid														
Eucalyptol*	0.56	(0.60)	0.19	(0.04)	0.13	(0.07)	0.44	(0.35)	0.19	(0.18)	0.13	(0.11)	0.88	(0.98)
(*Z*)-Linalool oxide*	0.21	(0.19)ab	0.47	(0.45)ab	0.77	(0.41)a	0.28	(0.30)ab	0.10	(0.17)b	0.26	(0.2)ab	0.58	(0.02)ab
Linalool*	1.1	(0.56)c	3.5	(1.7)b	3.1	(2.1)b	0.95	(0.75)c	1.4	(0.08)bc	1.6	(0.96)bc	11	(2.8)a
β-Damascenone**	42	(55)	38	(32)	60	(44)	38	(37)	8.3	(10)	61	(35)	10	(11)
Acids														
Hexanoic acid*	56	(21)a	51	(6)ab	34	(9)b	52	(12)a	38	(12)ab	42	(21)ab	36	(10)ab

Table S3. *Cont.*

2015	ADL [b]	BV	CV	EV	LC	RVL	MV	2015	ADL [b]	BV	CV	EV	LC	RVL
Carbonyls														
Hexanal*	60	(52)	47	(23)	33	(18)	51	(27)	41	(6.3)	62	(74)	36	(13)
(*E*)-2-Hexenal*	97	(43)ab	130	(106)	71	(19)bc	69	(44)bc	44	(5)bc	36	(16)c	57	(5)bc
2-Octanone*	0.41	(0.24)	0.34	(0.10)	0.34	(0.04)	0.48	(0.19)	0.38	(0.16)	0.41	(0.18)	0.52	(0.14)
Nonanal*	3.1	(1.9)a	2.4	(1.2)ab	1.8	(0.5)b	3.3	(1.3)a	2.0	(0.5)ab	2.3	(1.8)ab	2.7	(0.5)ab
Isophorone**	3.3	(1.3)	3.1	(0.9)	2.7	(0.4)	3.2	(0.8)	3.2	(0.1)	2.7	(1.0)	3.5	(0.1)
Benzaldehyde**	4.4	(1.3)ab	3.2	(1.7)ab	2.8	(0.6)b	4.7	(1.6)a	5.9	(2.1)a	3.3	(0.8)ab	5.0	(1.1)a
**2016**														
Ethyl esters														
Ethyl pentanoate*	5.6	(1.7)	7.8	(2.0)	7.9	(1.5)	7.9	(2.6)	5.8	(2.0)	7.4	(2.4)	- [d]	
Ethyl hexanoate*	1.0	(0.3)	1.9	(0.9)	1.6	(1.0)	1.2	(0.4)	1.6	(1.2)	2.0	(1.5)	-	
Ethyl octanoate*	0.46	(0.23)b	1.1	(0.52)a	0.67	(0.26)ab	0.46	(0.12)b	0.47	(0.18)b	0.68	(0.22)ab	-	
Diethyl succinate**	0.036	(0.016)	0.055	(0.032)	0.050	(0.016)	0.032	(0.010)	0.030	(0.011)	0.050	(0.012)	-	
Acetate esters														
Isoamyl acetate*	3.6	(0.4)	3.6	(1.8)	3.0	(0.7)	3.3	(0.5)	2.9	(0.8)	4.5	(0.8)	-	
Hexyl acetate*	1.5	(0.7)ab	1.9	(0.2)a	1.0	(0.4)b	1.1	(0.4)ab	1.4	(0.5)ab	1.6	(0.7)ab	-	
Alcohols														
3-Methyl-1-butanol*	5.4	(1.6)	1.9	(0.6)	5.9	(4.7)	5.9	(2.7)	4.8	(2.7)	2.3	(1.4)	-	
1-Hexanol*	236	(32)	213	(53)	180	(28)	239	(79)	240	(44)	225	(34)	-	
(*E*)-3-Hexen-1-ol*	0.68	(0.17)	0.93	(0.29)	0.68	(0.15)	0.82	(0.32)	0.68	(0.25)	0.59	(0.13)	-	
(*Z*)-3-Hexen-1-ol*	14	(3)ab	12	(3)ab	18	(6)ab	25	(12)a	10	(2)b	12	(2)ab	-	
(*E*)-2-Hexen-1-ol*	107	(21)	169	(45)	132	(22)	167	(76)	119	(35)	145	(33)	-	
(*Z*)-2-Hexen-2-ol*	1.8	(0.6)a	2.5	(0.7)a	1.9	(0.4)a	2.5	(1.4)a	1.5	(0.4)a	1.6	(0.6)a	-	
1-Octen-3-ol*	10	(3.2)	7.5	(3.0)	7.9	(3.1)	9.2	(3.8)	6.5	(1.8)	7.0	(2.1)	-	
2-Ethyl-1-hexanol*	2.4	(0.4)b	4.9	(1.6)a	4.5	(1.6)a	2.9	(0.4)b	2.8	(0.4)b	5.4	(2.0)b	-	
1-Octanol*	0.98	(0.36)	1.0	(0.2)	1.0	(0.2)	1.0	(0.3)	0.66	(0.20)	0.91	(0.16)	-	
2-Phenylethanol**	89	(15)a	23	(6)c	39	(7)bc	65	(24)ab	38	(2)bc	58	(8)b	-	
Benzyl alcohol**	47	(11)a	10	(3)c	18	(4)bc	24	(9)b	15	(2)bc	31	(10)b	-	

Table S3. *Cont.*

2016	ADL [b]	BV	CV	EV	LC	RVL	MV	2015	ADL [b]	BV	CV	EV	LC	RVL
Isoprenoid														
Eucalyptol*	0.065	(0.026)	0.49	(0.9)	0.19	(0.2)	0.095	(0.034)	0.041	(0.034)	0.19	(0.08)		-
(*Z*)-Linalool oxide*	0.26	(0.10)ab	0.33	(0.34)ab	0.43	(0.17)a	0.21	(0.13)b	0.06	(0.06)b	0.15	(0.02)b		-
Linalool*	0.91	(0.37)b	1.8	(1.5)ab	3.0	(1.1)a	1.4	(1.2)b	0.88	(0.4)b	0.65	(0.14)b		-
β-Damascenone**	22	(24)	18	(28)	25	(25)	26	(20)	3	(3)	26	(21)		-
Acids														
Hexanoic acid*	62	(14)a	56	(13)ab	43	(17)ab	48	(15)ab	52	(10)ab	35	(6)b		-
Carbonyls														
Hexanal*	68	(51)	14	(6)	65	(64)	68	(32)	68	(64)	29	(13)		-
(*E*)-2-Hexenal*	149	(34)	136	(60)	186	(91)	195	(62)	159	(39)	110	(30)		-
2-Octanone*	0.46	(0.16)ab	0.70	(0.05)a	0.57	(0.16)ab	0.41	(0.09)b	0.45	(0.23)ab	0.67	(0.10)a		-
Nonanal*	1.6	(0.5)b	3.3	(0.7)a	2.3	(0.8)ab	2.1	(0.9)b	1.4	(0.4)b	1.4	(0.2)b		-
Isophorone**	2.2	(0.4)	2.2	(0.3)	2.8	(0.9)	3.3	(1.0)	2.2	(0.6)	2.2	(0.4)		-
Benzaldehyde**	2.5	(0.5)	2.2	(0.2)	2.3	(0.4)	2.0	(0.4)	2.0	(0.2)	2.2	(0.1)		-

[a] For each region, means ± SD (duplicate measurements for each sample) with different letters within a row (a, b, c) are significantly different ($p < 0.05$) according to Tukey's (HSD) pairwise comparison. [b] Adelaide Hills (ADL, $n = 8$), Barossa Valley (BV, $n = 5$), Clare Valley (CV, $n = 9$), Eden Valley (EV, $n_{2015} = 15$ and $n_{2016} = 12$), Langhorne Creek (LC, $n = 3$), Riverland (RVL, $n = 4$), McLaren Vale (MV, $n = 2$). [c] Values expressed as mg/L equivalents of *d_{13}-1-hexanol or **d_5-2-phenylethanol. [d] -, not sampled.

Table S4. Mean content (mg/L) of amino acids in Chardonnay berries at harvest from seven Geographical Indications in South Australia in 2015 and 2016. [a]

2015	ADL [b]		BV		CV		EV		LC		RVL		MV	
Aspartic acid	26	(8.5)a	29	(14)a	12	(3.9)b	25	(6.2)a	19	(10)ab	25	(6.7)ab	9.6	(2.8)b
Asparagine	26	(11)	35	(23)	16	(9.2)	29	(6.8)	41	(24)	28	(14)	20	(8.9)
Serine	113	(23)ab	115	(46)ab	72	(15)c	133	(23)a	84	(16)bc	88	(4.7)bc	51	(9.2)c
Glutamic acid	59	(19)a	60	(27)a	26	(9.4)b	59	(13)a	52	(10)ab	59	(17)a	36	(7.6)ab
Histidine	60	(20)ab	62	(29)ab	35	(11)b	72	(17)a	56	(5.6)ab	62	(22)ab	33	(10)b
GLN + GLY	267	(80)a	265	(213)a	99	(55)b	335	(66)a	161	(104)ab	265	(49)ab	53	(3.4)b
Arginine	370	(111)ab	457	(169)ab	301	(136)b	485	(61)a	454	(52)ab	456	(66)ab	225	(88)b
Threonine	87	(21)ab	100	(30)ab	78	(15)b	118	(26)a	96	(3.4)ab	81	(3.8)b	64	(19)b
β-Alanine	22	(2.1)bc	24	(2.7)ab	20	(2.5)c	24	(2.5)b	29	(0.8)a	25	(1.0)ab	24	(2.3)ab
Alanine	264	(29)ab	227	(72)b	148	(55)c	307	(28)a	201	(27)bc	253	(24)ab	103	(10)c
Proline	631	(157)b	749	(154)ab	454	(164)b	749	(127)ab	959	(111)a	794	(130)ab	803	(209)ab
γ-Aminobutyric acid	217	(30)ab	223	(41)ab	168	(31)b	239	(40)a	256	(23)a	226	(33)ab	181	(64)ab
Tyrosine	7.7	(2.6)ab	9.8	(4.1)a	4.1	(1.1)b	6.4	(2.7)ab	6.3	(1.2)ab	5.8	(1.2)ab	5.0	(1.0)ab
Valine	38	(16)ab	32	(11)ab	25	(6.6)b	47	(13)a	39	(4.3)ab	32	(0.7)ab	23	(7.3)ab
Methionine	6.6	(3.6)	5.2	(2.2)	3.4	(0.6)	7.1	(4.0)	3.7	(0.5)	4.3	(0.8)	4.2	(0.3)
Lysine	8.3	(2.9)	10	(3.1)	8.2	(3.0)	9.5	(2.4)	10	(2.1)	11	(1.5)	6.4	(2.7)
Isoleucine	17	(12)	15	(5.6)	14	(4.3)	28	(19)	20	(3.2)	13	(0.5)	12	(4.9)
Leucine	24	(12)	26	(10)	19	(4.5)	35	(19)	34	(5.4)	22	(0.9)	19	(6.5)
Phenylalanine	29	(13)	25	(8.1)	20	(6.2)	36	(18)	33	(4.6)	25	(3.1)	23	(9.1)
**2016**														
Aspartic acid	65	(13)ab	28	(16)c	29	(9.4)c	40	(13)c	42	(10)bc	84	(14)a	- [c]	
Asparagine	15	(3.6)	11	(2.8)	17	(16)	18	(11)	27	(6.6)	15	(6.4)	-	
Serine	127	(15)a	105	(41)ab	84	(26)b	120	(41)ab	80	(5.4)b	88	(9.3)ab	-	
Glutamic acid	183	(54)a	141	(34)ab	122	(12)b	161	(37)ab	151	(20)ab	156	(11)ab	-	
Histidine	63	(13)	70	(29)	65	(25)	74	(35)	47	(6.3)	51	(12)	-	
GLN + GLY	246	(107)a	99	(51)b	117	(77)b	158	(93)ab	98	(28)b	218	(105)ab	-	
Arginine	510	(130)	412	(186)	369	(183)	468	(225)	384	(51)	344	(150)	-	
Threonine	116	(8.6)	105	(27)	95	(30)	118	(36)	90	(2.3)	75	(6.3)	-	
β-Alanine	20	(1.9)	18	(1.1)	19	(1.6)	20	(1.6)	20	(1.7)	18	(1.6)	-	
Alanine	249	(48)a	120	(65)c	130	(68)c	150	(51)bc	136	(31)bc	231	(34)ab	-	
Proline	784	(130)a	445	(161)b	364	(160)b	512	(203)b	657	(119)ab	330	(82)b	-	
γ-Aminobutyric acid	109	(62)	104	(38)	102	(38)	93	(24)	96	(11)	58	(12)	-	
Tyrosine	17	(1.8)	18	(6.4)	16	(6.0)	21	(8.1)	13	(1.5)	14	(2.5)	-	
Valine	31	(2.7)	25	(11)	28	(16)	32	(10)	25	(1.1)	22	(1.6)	-	

Table S4. *Cont.*

2016	ADL [b]	BV	CV	EV	LC	RVL	MV	2015	ADL [b]	BV	CV	EV	LC	RVL
Methionine	9.4	(3.9)b	18	(4.1)a	13	(6.6)ab	8.2	(3.0)b	7.0	(4.6)b	20	(1.8)a		-
Lysine	8.0	(1.1)	9.1	(3.5)	8.1	(2.7)	9.0	(3.1)	8.3	(1.1)	6.6	(1.6)		-
Isoleucine	15	(1.9)	14	(5.9)	18	(13)	20	(8.6)	14	(0.7)	12	(1.3)		-
Leucine	23	(2.8)	20	(7.7)	22	(13)	25	(8.6)	21	(0.8)	13	(1.9)		-
Phenylalanine	24	(4.0)	23	(6.0)	24	(11)	32	(10)	27	(4.6)	18	(1.6)		-

[a] For each region, means ± SD (duplicate measurements for each sample) with different letters within a row (a, b, c) are significantly different ($p < 0.05$) according to Tukey's (HSD) pairwise comparison. [b] Adelaide Hills (ADL, n = 8), Barossa Valley (BV, n = 5), Clare Valley (CV, n = 9), Eden Valley (EV, n_{2015} = 15 and n_{2016} = 12), Langhorne Creek (LC, n = 3), Riverland (RVL, n = 4), McLaren Vale (MV, n = 2). GLN + GLY, Glutamine and glycine. [c] -, not sampled.

Table S5. Mean concentrations (expressed as mg/L of deuterated internal standard) of hydrolytically-released volatile compounds determined in harvest samples of Chardonnay berries collected from seven Geographical Indications within South Australia in 2015 and 2016. [a]

2015	ADL [b]		BV		CV		EV		LC		RVL		MV	
Alcohols														
3-Methyl-1-butanol	0.52	(0.13)	0.48	(0.23)	0.53	(0.11)	0.62	(0.26)	0.74	(0.21)	0.46	(0.21)	0.68	(0.16)
Benzyl Alcohol** [c]	1.8	(0.44)ab	2.6	(1.9)ab	1.3	(0.36)b	2.9	(1.9)ab	4.0	(1.8)ab	2.1	(1.1)ab	4.7	(0.66)a
2-Phenylethanol**	2.0	(0.5)c	3.0	(2.1)bc	1.5	(0.4)c	3.1	(2.0)bc	5.4	(2.3)ab	1.3	(0.2)c	6.9	(1.6)a
Isoprenoids														
(*E*)-Linalool oxide*	1.4	(0.35)	1.5	(0.87)	1.7	(0.37)	1.8	(0.88)	2.1	(0.68)	0.96	(0.66)	2.5	(1.2)
(*Z*)-Linalool oxide*	0.91	(0.26)	0.92	(0.58)	1.0	(0.26)	1.3	(0.59)	1.4	(0.42)	0.67	(0.42)	1.6	(0.79)
α-Terpinene*	0.062	(0.020)	0.020	(0.020)	0.042	(0.017)	0.047	(0.029)	0.047	(0.038)	0.041	(0.047)	0.052	(0.013)
Vitispirane (sum of isomers)*	0.20	(0.12)	0.089	(0.09)	0.31	(0.16)	0.27	(0.17)	0.43	(0.29)	0.13	(0.20)	0.40	(0.03)
Linalool*	0.066	(0.031)	0.042	(0.004)	0.068	(0.025)	0.066	(0.030)	0.057	(0.010)	0.031	(0.021)	0.055	(0.004)
α-Terpineol*	0.48	(0.14)b	0.49	(0.29)b	0.83	(0.26)ab	0.52	(0.18)b	0.61	(0.04)b	0.47	(0.37)b	1.4	(0.64)a
β-Damascenone*	0.81	(0.38)	0.54	(0.42)	0.95	(0.45)	1.2	(0.72)	1.6	(0.36)	0.64	(0.65)	1.8	(0.81)
TDN*	0.36	(0.22)c	0.43	(0.40)bc	0.21	(0.13)c	0.65	(0.53)bc	1.4	(0.89)ab	0.35	(0.51)c	2.2	(1.1)a
β-Ionone*	3.5	(0.67)	4.4	(1.3)	5.5	(1.3)	5.0	(1.5)	6.0	(2.2)	3.9	(1.5)	5.6	(1.5)
2,6-Dimethyl-7-octene-2,6-diol*	0.51	(0.17)ab	0.40	(0.11)b	0.81	(0.36)a	0.44	(0.17)b	0.41	(0.22)b	0.57	(0.20)ab	0.46	(0.02)ab
3-Oxo-α-ionol*	7.8	(1.1)b	11	(3.6)ab	12	(2.5)a	10	(2.3)ab	7.2	(1.2)b	8.9	(0.7)ab	6.6	(1.2)b

Table S5. *Cont.*

2015	ADL [b]	BV	CV	EV	LC	RVL	MV	2015	ADL [b]	BV	CV	EV	LC	RVL
Carbonyls														
Hexanal*	0.83	(0.31)	0.74	(0.57)	0.61	(0.30)	1.3	(0.83)	1.4	(0.55)	0.56	(0.20)	0.75	(0.14)
(*E*)-2-Hexenal*	1.0	(0.62)	0.70	(0.70)	0.72	(0.28)	0.98	(0.43)	1.3	(0.44)	0.61	(0.44)	0.80	(0.06)
5-Methyl furfural*	0.28	(0.08)b	0.58	(0.30)ab	0.40	(0.10)ab	0.46	(0.16)ab	0.58	(0.15)ab	0.46	(0.18)ab	0.63	(0.21)a
Phenylacetaldehyde**	0.64	(0.33)	0.40	(0.41)	0.37	(0.11)	0.86	(0.52)	0.90	(0.24)	0.26	(0.15)	0.48	(0.06)
Benzaldehyde*	0.84	(0.43)	0.89	(0.61)	0.52	(0.14)	1.1	(0.70)	1.5	(0.57)	0.66	(0.44)	1.4	(0.0)
Acetovanillone*	0.85	(0.20)	1.1	(0.64)	1.2	(0.43)	1.3	(0.54)	1.2	(0.35)	0.82	(0.14)	1.4	(0.56)
Acids														
Hexanoic acid*	24	(9.1)	15	(2.6)	24	(6.0)	18	(6.6)	12	(1.8)	21	(3.3)	9	(4.9)
Octanoic Acid*	1.2	(0.47)	0.98	(0.58)	1.0	(0.20)	1.7	(1.0)	1.5	(0.59)	1.0	(0.46)	1.4	(0.44)
Hexadecanoic acid*	4.4	(2.7)	4.9	(3.6)	3.3	(1.2)	5.6	(4.5)	5.4	(2.2)	3.1	(1.7)	6.1	(2.1)
Volatile phenols														
Guaiacol*	0.078	(0.026)	0.064	(0.018)	0.066	(0.016)	0.088	(0.053)	0.13	(0.026)	0.058	(0.016)	0.14	(0.086)
4-Vinylguaiacol*	7.8	(2.6)b	11	(5.5)ab	11	(4.9)ab	9.3	(3.9)b	11	(4.2)ab	10	(1.0)ab	20	(8.0)a
4-Allyl-2,6-dimethoxyphenol**	0.56	(0.19)	0.73	(0.27)	0.48	(0.21)	0.75	(0.39)	0.72	(0.16)	0.65	(0.17)	0.62	(0.10)
Vanillin**	0.44	(0.15)	0.56	(0.26)	0.49	(0.10)	0.54	(0.23)	0.58	(0.15)	0.33	(0.07)	0.63	(0.04)
Methyl vanillate**	0.87	(0.14)ab	0.82	(0.52)ab	0.48	(0.17)b	1.1	(0.49)a	0.95	(0.25)ab	0.77	(0.14)ab	0.50	(0.19)ab
2,6-Dimethoxyphenol*	0.12	(0.05)	0.13	(0.07)	0.093	(0.030)	0.14	(0.07)	0.13	(0.02)	0.11	(0.02)	0.17	(0.08)
**2016**														
Alcohols														
3-Methyl-1-butanol	0.16	(0.01)a	0.15	(0.03)ab	0.14	(0.04)ab	0.12	(0.03)b	0.11	(0.01)b	0.13	(0.04)ab		- [d]
Benzyl Alcohol**	2.1	(1.9)	1.6	(0.07)	2.3	(1.8)	1.4	(0.46)	1.5	(0.04)	1.5	(0.26)		-
2-Phenylethanol**	1.4	(0.82)	1.1	(0.18)	1.3	(0.81)	0.94	(0.44)	1.1	(0.07)	0.86	(0.17)		-
Isoprenoids														
(*E*)-Linalool oxide*	0.72	(0.27)a	0.52	(0.11)ab	0.56	(0.17)ab	0.37	(0.20)b	0.30	(0.02)b	0.39	(0.33)ab		-
(*Z*)-Linalool oxide*	0.51	(0.21)a	0.40	(0.07)ab	0.47	(0.12)a	0.26	(0.14)b	0.23	(0.03)b	0.31	(0.21)ab		-
α-Terpinene*	0.0071	(0.0026)a	0.0044	(0.0019)ab	0.0046	(0.0022)ab	0.0037	(0.0013)b	0.0056	(0.0058)ab	0.0039	(0.0020)ab		-
Vitispirane (sum of isomers)*	0.033	(0.011)b	0.058	(0.016)a	0.045	(0.019)ab	0.030	(0.009)b	0.019	(0.005)b	0.024	(0.017)b		-
Linalool*	0.015	(0.005)ab	0.021	(0.009)a	0.014	(0.008)ab	0.0086	(0.0052)b	0.0060	(0.0007)b	0.012	(0.008)ab		-
α-Terpineol*	0.26	(0.06)ab	0.38	(0.13)a	0.29	(0.09)ab	0.18	(0.10)b	0.16	(0.05)b	0.25	(0.17)ab		-
β-Damascenone*	0.24	(0.04)ab	0.27	(0.07)a	0.25	(0.08)a	0.16	(0.06)b	0.21	(0.12)ab	0.15	(0.03)b		-

Table S5. *Cont.*

**2016**	**ADL**[b]	**BV**	**CV**	**EV**	**LC**	**RVL**	**MV**	**2015**	**ADL**[b]	**BV**	**CV**	**EV**	**LC**	**RVL**
Isoprenoids														
TDN*	0.23	(0.15)	0.35	(0.21)	0.22	(0.13)	0.15	(0.19)	0.059	(0.011)	0.15	(0.13)		-
β-Ionone*	3.2	(1.2)bc	4.9	(1.3)ab	5.6	(2.0)a	2.8	(1.0)bc	2.6	(0.3)bc	1.9	(1.0)c		-
2,6-Dimethyl-7-octene-2,6-diol*	0.51	(0.10)ab	0.70	(0.19)a	0.53	(0.29)ab	0.29	(0.14)b	0.19	(0.03)b	0.42	(0.26)ab		-
3-Oxo-α-ionol*	8.0	(5.3)ab	6.2	(0.66)ab	12	(6.8)a	5.7	(1.3)b	7.4	(0.33)ab	5.0	(0.97)b		-
Carbonyls														
Hexanal*	0.51	(0.21)a	0.31	(0.08)ab	0.39	(0.27)ab	0.25	(0.14)b	0.31	(0.07)ab	0.34	(0.10)ab		-
(*E*)-2-Hexenal*	0.49	(0.17)a	0.43	(0.13)ab	0.38	(0.24)b	0.26	(0.11)b	0.20	(0.02)b	0.38	(0.21)ab		-
5-Methyl furfural*	0.32	(0.07)ab	0.49	(0.05)a	0.45	(0.16)a	0.26	(0.17)b	0.19	(0.03)b	0.28	(0.07)ab		-
Phenylacetaldehyde**	0.16	(0.03)	0.16	(0.07)	0.16	(0.09)	0.12	(0.07)	0.07	(0.01)	0.12	(0.11)		-
Benzaldehyde*	0.20	(0.04)	0.25	(0.08)	0.19	(0.06)	0.16	(0.05)	0.14	(0.08)	0.17	(0.05)		-
Acetovanillone*	0.76	(0.48)	0.50	(0.09)	0.87	(0.37)	0.58	(0.13)	0.68	(0.08)	0.50	(0.04)		-
Acids														
Hexanoic acid*	4.9	(1.7)a	2.1	(0.4)b	2.4	(1.1)b	1.9	(1.1)b	1.5	(0.2)b	2.2	(2.2)b		-
Octanoic Acid*	0.54	(0.16)a	0.32	(0.08)ab	0.29	(0.14)b	0.23	(0.14)b	0.10	(0.01)b	0.36	(0.20)ab		-
Hexadecanoic acid*	3.3	(2.1)	1.6	(0.4)	2.3	(1.6)	1.7	(0.8)	1.4	(0.7)	1.9	(0.4)		-
Volatile phenols														
Guaiacol*	0.11	(0.04)a	0.067	(0.010)ab	0.086	(0.040)ab	0.055	(0.028)b	0.054	(0.005)b	0.076	(0.034)ab		-
4-Vinylguaiacol*	25	(9.8)a	14	(2.2)ab	16	(11)ab	10	(9.3)b	6.2	(0.50)b	14	(9.0)ab		-
4-Allyl-2,6-dimethoxyphenol**	0.29	(0.07)ab	0.24	(0.05)ab	0.39	(0.31)a	0.15	(0.10)b	0.23	(0.04)ab	0.24	(0.07)ab		-
Vanillin**	0.21	(0.06)	0.21	(0.03)	0.21	(0.09)	0.11	(0.05)	0.22	(0.06)	0.18	(0.06)		-
Methyl vanillate**	0.50	(0.21)a	0.14	(0.02)b	0.29	(0.19)b	0.18	(0.10)b	0.35	(0.01)ab	0.28	(0.10)b		-
2,6-Dimethoxyphenol*	0.31	(0.15)a	0.10	(0.02)b	0.15	(0.09)b	0.10	(0.08)b	0.066	(0.005)b	0.17	(0.11)ab		-

[a] For each region, means ± SD (duplicate measurements for each sample) with different letters within a row (a, b, c) are significantly different ($p < 0.05$) according to Tukey's (HSD) pairwise comparison. [b] Adelaide Hills (ADL, $n = 8$), Barossa Valley (BV, $n = 5$), Clare Valley (CV, $n = 9$) and Eden Valley (EV, $n_{2015} = 15$ and $n_{2016} = 12$), Langhorne Creek (LC, $n = 3$), Riverland (RVL, $n = 4$), McLaren Vale (MV, $n = 2$). [c] Values expressed as mg/L equivalents of *d_{13}-1-hexanol or **d_5-2-phenylethanol. [d] -, not sampled.

Table S6. Weather data for all regions sampled, including mean, minimum, maximum, and highest temperature for the months of January and February, number of days when temperature exceeded 25 and 30 °C during the January-February period, GDD [a] values, and total rainfall and solar exposure for the months of January and February.

	Mean T (°C)		Highest T (°C)		No. of Days (°C)		GDD [a]	Rainfall (mm)		Solar Exposure (MJ/m²)	
	min/max	min/max									
	Jan	Feb	Jan	Feb	>25	>30		Jan	Feb	Jan	Feb
**2015**											
ADL [b]	12.5/25.8	13.7/29.8	41.2	39.6	37	20	1639	52.8	0.0	23.7	23.7
BV	15.9/28.7	16.2/33.4	43.6	41.2	50	28	2074	55.4	3.4	24.4	24.2
CV	14.7/27.4	15.3/32.3	40.8	39.5	45	26	1884	62.8	0.0	23.7	25.1
EV	13.2/25.0	14.7/29.6	40.0	37.2	35	23	1576	89.2	1.0	23.6	24.2
LC	14.9/26.5	15.0/29.2	43.5	39.4	32	18	1839	66.2	0.8	23.5	22.3
MV	17.9/27.0	17.7/30.4	42.3	40.0	39	22	2086	33.8	0.2	24.0	23.9
RVL	15.7/31.4	15.6/35.2	45.1	44.1	57	40	2307	31.6	0.0	25.5	25.4
**2016**											
ADL	13.9/29.1	13.3/26.6	38.7	36.1	41	18	1998	35.5	37.6	25.6	23.5
BV	16.3/31.8	13.1/29.6	39.9	37.2	53	34	2406	30.8	5.4	25.5	24.6
CV	15.7/30.2	13.8/29.5	38.1	38.2	47	29	2227	35.8	18.0	24.6	24.8
EV	15.2/28.0	13.2/26.8	36.6	36.4	39	22	1985	30.8	27.8	25.7	24.6
LC	15.3/28.2	14.6/26.0	37.2	37.0	34	16	2101	17.0	19.6	25.1	22.0
MV	17.9/28.9	16.3/26.6	37.2	37.5	35	19	2388	29.2	44.6	26.3	22.9
RVL	16.4/33.9	14.7/33.6	43.3	44.6	56	43	2670	21.0	0.0	25.8	25.4

[a] Growing degree days base 10 °C. [b] ADL, Adelaide Hills; BV, Barossa Valley; CV, Clare Valley; EV, Eden Valley; LC, Langhorne Creek; RVL, Riverland; MV, McLaren Vale.
